# Collective Molecular Machines: Multidimensionality and Reconfigurability

**DOI:** 10.1007/s40820-024-01379-4

**Published:** 2024-03-18

**Authors:** Bin Wang, Yuan Lu

**Affiliations:** 1 Department of Chemical Engineering, Tsinghua University, Beijing, 100084 People’s Republic of China; 2 Key Laboratory of Industrial Biocatalysis, Ministry of Education, Tsinghua University, Beijing, 100084 People’s Republic of China

**Keywords:** Molecular machines, Collective control, Collective behaviors, DNA, Biomolecular motors

## Abstract

Molecular machines are key to cellular activity where they are involved in converting chemical and light energy into efficient mechanical work. During the last 60 years, designing molecular structures capable of generating unidirectional mechanical motion at the nanoscale has been the topic of intense research. Effective progress has been made, attributed to advances in various fields such as supramolecular chemistry, biology and nanotechnology, and informatics. However, individual molecular machines are only capable of producing nanometer work and generally have only a single functionality. In order to address these problems, collective behaviors realized by integrating several or more of these individual mechanical units in space and time have become a new paradigm. In this review, we comprehensively discuss recent developments in the collective behaviors of molecular machines. In particular, collective behavior is divided into two paradigms. One is the appropriate integration of molecular machines to efficiently amplify molecular motions and deformations to construct novel functional materials. The other is the construction of swarming modes at the supramolecular level to perform nanoscale or microscale operations. We discuss design strategies for both modes and focus on the modulation of features and properties. Subsequently, in order to address existing challenges, the idea of transferring experience gained in the field of micro/nano robotics is presented, offering prospects for future developments in the collective behavior of molecular machines.

## Highlights

- Recent advances and design strategies for molecular machines working as collectives in building smart responsive materials and micro/nanoscale operations are summarized in this review.
- The modulation of collective behaviors characteristics and properties is summarized in focus, including reversibility, amplification, anisotropy and reconfigurability of smart materials, as well as reconfigurability, orthogonality and logical control of swarming.
- Experiences and paradigms in the field of micro/nanorobotics in collective construction, control strategies, and model transformations are expected to provide guidance for molecular machines to build reconfigurable, multi-dimensional, and multi-modularity advanced collectives.

## Graphical abstract

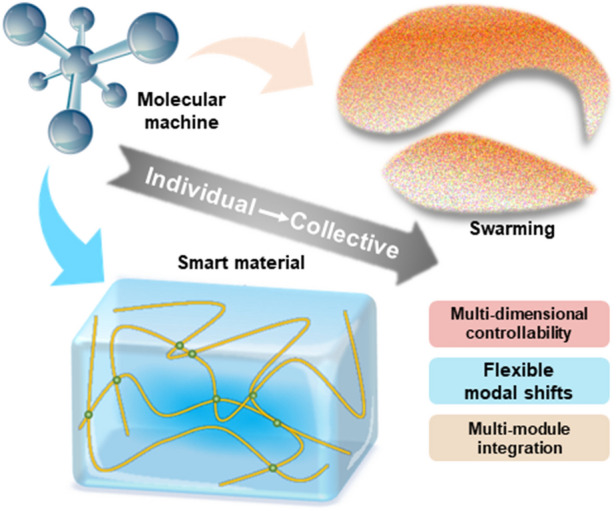

## Introduction

One of the common definitions of a machine is a device that contains moving parts that are induced by some kind of energy source to move relative to each other to accomplish a specific task. Similarly, molecular machines are nanoscale molecular or supramolecular systems capable of generating mechanical motion, such as translational, rotational, or conformational motion. The concept of "molecular machines" is different from "molecules" and "molecular rotors". Molecular machines and molecular rotors are both a special class of molecules that can produce certain conformational changes. The difference is that conformational changes in molecular rotors tend to be free, such as rotation of single bonds or rapid and reversible switching between the two conformations in thermal equilibrium, which means that no effective work can be produced. Molecular machines, on the other hand, can be thought of as special molecular rotors that convert heat, chemical, or light energy into directed isomerization. This transformation is controllable, just as a robotic arm can be controlled in the macro world, and can therefore produce effective work. The potential of molecular machines is attractive [1], because they can be used as robotic arms to change or create the molecular world. The minimum requirements for a molecular machine are that it must 1) consume energy, 2) have moving parts, and 3) complete the task [2]. It is worth noting that some researchers like to refer to molecular machines as "molecular robots." The truth is that although these artificial molecular machines have met the three most basic requirements, they are not yet able to work autonomously compared to natural molecular machines. Therefore, in this sense, "molecular robot" is more suitable to call natural molecular machines, and is not quite appropriate for artificial molecular machines. For ease of elaboration, both are referred to as "molecular machines" in this review.

Molecular machines are inspired by the imitation of living systems. Natural molecular machines, dominated by motor proteins, direct specific molecular conformational changes by expending energy and are key to almost all fundamental processes, from transport to cell division and ATP production [3]. The design and synthesis of artificial molecular systems capable of precise mechanical actuation and energy conversion must begin with a fine-grained understanding of the thermodynamic parameters that govern at the nanoscale [4]. It is impossible to extract useful mechanical work at the nanoscale from simple molecular motions without introducing the necessary elements to correct for thermal fluctuations. This is because individual molecules usually obey Brownian motion. This was a concept introduced by Richard Feynman 60 years ago. With advances in synthetic chemistry, supramolecular chemistry, nanotechnology, and biomolecular engineering, a variety of strategies through manipulation of nonequilibrium energy barriers have been proposed, enabling molecular machines to produce directed motion and controlled transformations [5–13]. A variety of organic small molecules and supramolecular systems (e.g., helicenes and rotaxanes), as well as molecular machines based on naturally occurring biological macromolecules (e.g., DNA/RNA nanostructures) [14–20] and biomolecular proteins [21–24] have been developed and proved to be very promising for performing operations at the nanoscale, e.g., microscopic surface modification [25–27], transmembrane transport [28–30] and smart catalysis [31, 32]. In general, the functional tasks that can be performed using these machines have been carried out at their own scale. However, the limited nanoscale work makes molecular machines require new trends of integrating and connecting with other elements to produce collective behavior.

What new paradigm will collective behavior bring to molecular machines? Looking back at living systems with this question in mind, one conclusion becomes obvious; living systems tend to utilize molecular machines as a whole to perform tasks. In the mode of execution, through clever coupling with the environment, molecular movements are integrated and amplified, ultimately supporting large-scale functions such as cell swelling, muscle contraction, and cilia beating. In the mode of organization, they are divided into distinct work modules that are organized into highly ordered swarm factories through signals of high orthogonality. These ordered integrations and hierarchical organizations provide the inspiration to build meso- and macro-living materials and devices with autonomy and intelligence. The collective paradigm not only extends the scale of molecular machines from the nanoscale to the microscopic or even macroscopic scale, but also gains more degrees of freedom to perform more complex tasks. At this stage, two distinct but complementary collective paradigms have made remarkable research progress. On the one hand, a series of very fruitful efforts are directed by a research community strongly influenced by polymer chemists and materials scientists. They are building responsive materials and devices by appropriately integrating molecular machines into networks of other materials to efficiently amplify molecular motion and deformation. On the other hand, a series of breakthroughs have been made by another research community that is largely influenced by synthetic organic chemists and inspired by biological machines. They have constructed swarming patterns at the supramolecular level. These molecular machines can be assigned complex molecular tasks that can be exploited through synergies in time and space, diversifying what would otherwise be nanoscale or microscale operations. The development of these two collective modes could greatly contribute to the matter synthesis, catalysis, biomedical and soft robotics industries.

To date, despite the large amount of work reported on the integration of molecular machines with soft matter, one-dimensional transformations, such as bending or stretching, still dominate. Multidimensional multimodal transformations need to be realized to move to truly intelligent materials. At the supramolecular level, some studies have been reported on the use of DNA and kinesin cluster behavior to perform tasks, but the complexity of the motion and the precision of the control are far from adequate compared to the modularity and integration of living systems. In the field of micro/nanorobots, collective behavior has been studied much earlier and more fruitfully. Reconfigurable, multidimensional and multimodule collective behaviors have all been reported. Although there are significant differences in scale as well as in the mechanisms of motion (molecular machines mainly through conformational and conformational changes, micro/nano robots mainly through physical field forces, local electric fields and local solute gradients), experiences and paradigms in collective construction, control strategies and mode shifts can provide guidance for molecular machine collective behaviors.

**Fig. 1:**
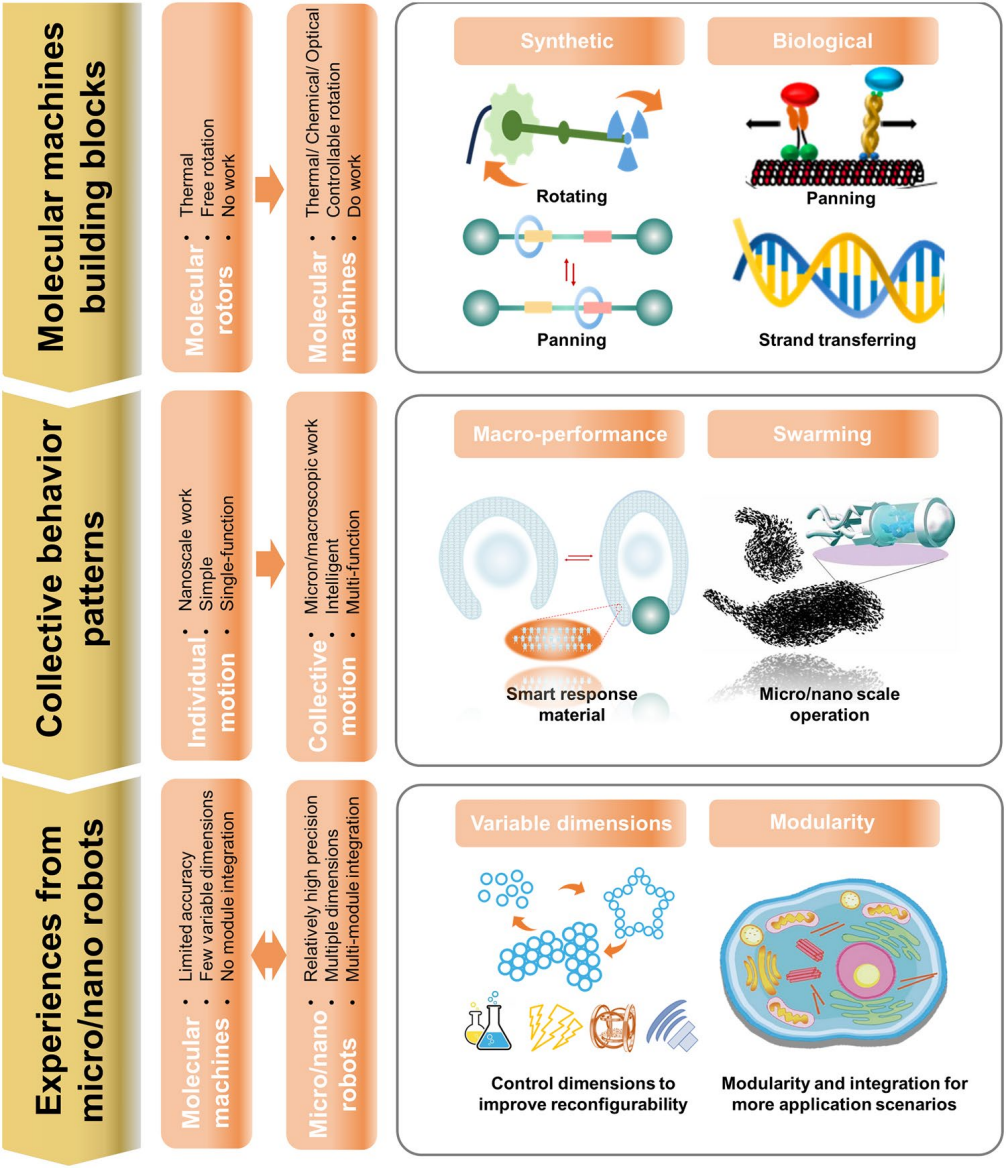
Synthetic and biological molecular machines are capable of converting energy to produce unidirectional conformational changes. They constitute the building blocks of collective behavior. There are two typical patterns of collective behavior, one that produces macro-behavior and the other that swarms. They are able to amplify the work and exhibit intelligence and versatility. The experience and paradigm of micro/nanorobots may be able to guide the design of collective behaviors of molecular machines to form multi-dimensional and multinodular

This paper discusses design strategies for the collective behavior of molecular machines, starting from the main fundamental building blocks, with a focus on summarizing recent advances in the coupling and control of collective modes at two different scales. Subsequently, important challenges that need to be addressed for the collective control of molecular machines as well as directions for development are discussed. To address these challenges, the idea of transferring experience gained in the field of micro/nanorobots is presented (Fig. 1).

## Basic Building Blocks of Molecular Machines

Unlike the macroscopic world, molecular machines are always in low Reynolds number environments with negligible momentum and inertial forces due to their small size [33], and thermal motion is the main factor dominating the state switching that occurs in molecular machines. Typical switching of molecular conformations or configurations can then be developed as molecular switches. When such transformations can be performed in a continuous cycle, they can be called molecular motors. The key issue here is how to make this transition unidirectional, which is the key to moving from a "molecule" to a "molecular machine". The necessary energy input is required to shift the conformation away from chemical equilibrium, while at the same time preventing the transition from proceeding in the opposite direction. This strategy guided the development of the ratchet mechanism, which led to the first rotating molecular machines. When scientists would synthesize mechanically interlocking molecules, the second type of translational molecular machine was greatly developed. By creating energy barriers, this molecular machine was able to switch controllably between different binding sites. This moves on a trajectory like a ribosome. While synthetic chemists worked on this, protein engineering and DNA origami techniques opened up new opportunities for molecular machines. Hybrid molecular machines based on biological macromolecules, such as motor proteins and DNA, were developed. The inherently delicate structure of biomolecular machines and their ability to utilize ATP reduced the design requirements. These three classes of molecular machines demonstrate the classical paradigm of locomotion and control are the basic building blocks for the formation of collective behavior. Here, their characteristics and principles are briefly discussed.

**Fig. 2:**
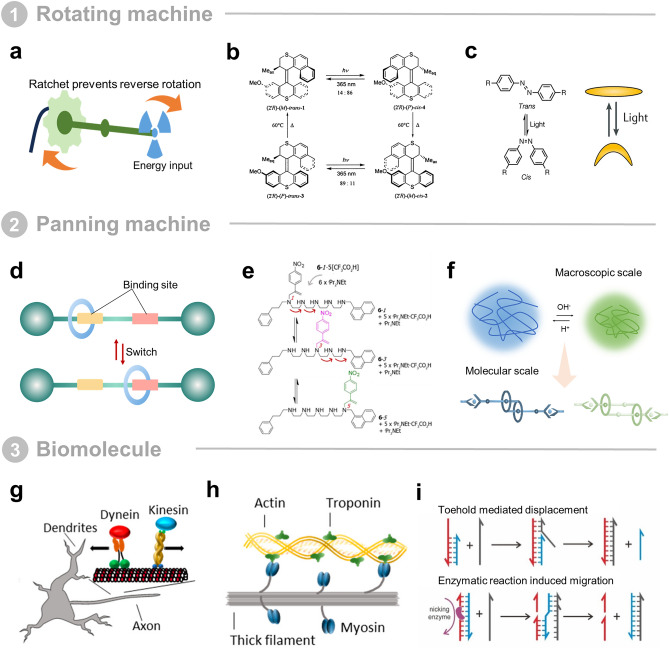
Main types of molecular machines. **a** Rotating molecular machines utilize external energy to produce rotation and prevent this rotation from being reversed by a ratchet mechanism. **b** Molecular motor with a different upper and lower part. The upper rotating unit repeats 360° rotation in four different steps under light of appropriate wavelengths and shows that the energy barrier of the thermal step in the rotational motion can be tuned by structural modifications. Reprinted permission from Ref. [34]. Copyright 2000, American Chemical Society. **c** Azobenzene molecular switch capable of producing *cis* and *trans* transitions under light. **d** The translational molecular machinery is able to migrate between different active sites. **e** Rearrangement of α-methylene-4-nitrostyrene from one amino group to another. Reprinted permission from Ref. [35]. Copyright 2013, American Chemical Society. **f** pH-switchable [c2] daisy chain. It mimics the ability of a muscle to produce contraction and stretch. **g** Kinesin and Dynein produce movement along microtubules. They are in opposite directions. Reprinted permission from Ref. [36]. Copyright 2018, American Chemical Society. **h** Actin, myosin and troponin make up the microstructure of muscle. Reprinted permission from Ref. [36]. Copyright 2018, American Chemical Society. **i** Two modes of strand migration of DNA, one toehold mediated and the other mediated by an enzymatic reaction. Reprinted permission from Ref. [37]. Copyright 2022, American Chemical Society

Rotating machines are commonplace in the macroscopic world, such as wheels and drills (Fig. 2a). Rotating motors also exist in nature, such as ATP synthases and bacterial flagella [38]. Such rotating machines share a common feature in that they all express their rotations continuously in a unidirectional manner [2]. In simple building blocks, such as rotations around single bonds and sandwich-like π-metal complexes [39–41], the most critical problem in realizing unidirectional rotations is to get the molecule out of its equilibrium state. This can be achieved by creating a combination of spatial site resistance and energy input. With this design philosophy in mind, much pioneering work has been accomplished. For example, in 1999, in Kelly's work, a monoamine-functionalized triptycene is connected by means of a single bond to a helicene [42]. After a whole process, the triptycene rotates unidirectionally in a clockwise manner relative to the helicene by approximately 120°. Feringa designed a light-driven unidirectional molecular rotor based on an overcrowded olefin. The combination of axial chirality and two stereocenters ensured that the four steps constituted a complete rotation of 360° in one direction only [43]. Subsequently, they developed a new type of molecular motor with distinct upper and lower half features (Fig. 2b) [34]. This is essential for coupling the rotor unit to a unidirectional rotating surface controlled by a single stereocenter. In subsequent work, the biaxially based rotor [44–47], the transitionally overcrowded olefin-based rotor [34, 48] and the imine-based rotor [49] were successively developed and improved. Rotating molecular machines have been used to design propellers [50], to reverse the enantioselectivity of asymmetric catalysts [31], and to move nano-cars freely on metallic copper surfaces [51]. When complete rotation is not required but only angular bending motions are needed, due to sensitivity to photoisomerization, azobenzene [52–56], stilbene [57–60], diaromatic ethylene [61–64] and spiropyran [65–69] have been incorporated into the molecular switch (Fig. 2c). Due to the advantages of being fast, reversible and easy to control, the optical modulation of such molecular machines can be easily integrated to perform collective behaviors.

The second type of rectilinear molecular machine enables the act of moving from one site to another, an active movement that results from a conformational change in the molecule or a difference in activity between sites (Fig. 2d). Linear molecular motors dominate in biological systems, for example, kinesins on microtubules (MTs) transport vesicles within cells and myosin is able to walk along actin filaments. Artificial organic small molecular machines also mimic this form of linear motion, although the way in which the motion is generated is different from the conformational change of the protein, and is achieved by the rearrangement of α-methylene-4-nitrostyrene from one amino group to the next (Fig. 2e) [35]. The incorporation of topology and the development of supramolecular chemistry have been key enablers of such molecular machines. For example, molecular shuttling and molecular switching based on bicycloalkanes and rotaxanes have been reported [70–72]. By differing in the strength of the recognition sites, the whole system is made to break the original equilibrium state, thus switching from one to the other between two different states. Building on these efforts, Leigh designed a fully synthetic molecular machine that mimics the function of the ribosome with which to synthesize sequence-defined peptides [73, 74]. Buhler and Giuseppone designed a pH-switchable [c2] daisy chain with terpyridine end groups (stoppers) to mimic muscle stimulus-responsive fibers (Fig. 2f). The [c2] daisy chain can be driven by changes in pH, producing differences in polymer chain lengths of more than 6 μm [75]. This work takes an important step forward by combining molecular machines with polymer chains, amplifying this amplification between molecules and microscopic mechanisms to the macroscopic to produce mechanical work, as they can self-assemble to form bundles [2]. In addition, these interlocked structure molecular machines have great potential in stimuli-responsive drug delivery, bioorthogonal catalysis, imaging, and cell membrane permeabilization [76].

The above two artificial molecular machines demonstrate the subtle design thinking of synthetic chemists. Natural biomolecular machines, on the other hand, already have more subtle structural design and the ability to utilize ATP under the guidance of the central law. Processing on this basis has clear advantages. Biomolecular motors are proteins responsible for mechanical movement in living organisms [77]. In most engineering applications, linear motors such as kinesin-1 and myosin II are predominant, with occasional use of dynein (Fig. 2g, h). The main role of kinesin-1 is to facilitate the transport of molecular cargo along MTs from the center of the cell to the periphery. Kinesin has a step length of 8 nm, can take more than 100 steps per second, and has an energy conversion efficiency of more than 40% [78, 79]. Compared to the kinesin-1 motor, dynein moves about 10 times slower, but in the opposite direction along the MT [80–83]. MT structures are self-assembled from α,β-tubulin heterodimers with an outer diameter of 25 nm and a length of typically a few micrometers [84]. The length of MTs in vitro can be altered by polymerization conditions. Myosin II is able to move along actin filaments and is responsible for muscle contraction. Similar to kinesin, myosin converts ATP into mechanical work and can generate forces of up to 9 pN and velocities of 15 μm s^−1^ in vitro [85–87]. These protein molecular motors are very efficient at converting chemical energy into mechanical work, and have therefore also been used as important motor modules in artificial molecular machines [22–24, 77]. Some groups have put considerable effort into synthesizing artificial motors based on proteins. For example, a synthetic protein motor called "tumbleweed" has been reported that is able to move along a linear track [88]. Another important class of biological macromolecules, DNA, is active in the realm of molecular machines. Due to unique chemical and physical properties, DNA plays an attractive role in molecular machines [89]. Firstly, the precise base complementary pairing principle of the DNA double helix ensures chemical stability along with high molecular recognition ability [90]. In performing a linear-like motion, the DNA walker is able to move accurately from one binding site to the next (Fig. 2i). Toe-mediated and enzymatic reaction-induced DNA strand displacement are the two main commonly used strategies [91–93]. In addition, DNA single strands are flexible polymers, whereas double-helical DNA structural domains are quite robust [94, 95]. Accompanied by the development of DNA origami technology, a wide range of localized stiffnesses can be achieved through the combination of flexible and rigid elements to meet the structural and functional requirements of any given design in two- dimension (2D) or three- dimension (3D) [96, 97]. Unlike any other material system, the predictable and guided folding of DNA is capable of manipulating molecular-scale processes and can therefore be combined with algorithms to create engineered and purposeful systems used as control molecular robots as dynamic processors [90, 98–104]. But they usually exhibit very low speeds [105], more predominantly used as auxiliary units in molecular machines, facilitating swarming through the polymerization and depolymerization of double strands making it possible to mediate interactions between moving molecular robots.

Molecular machines are gradually becoming an active area of research based on the above building blocks for controlled movement. Applications at the microscopic scale, such as oligomer synthesis [74, 106], product chiral switching [31, 107], molecular transport [108–110], biosensing [111–114] have also been studied. Nobel Laureate Professor J. Fraser Stoddart argues that there is certainly scope for molecular machines to find applications at all length scales, but we do need to invest more time and effort in investigating how to harness and amplify their output [2].

## Construction Strategies for Collective Molecular Machines

Despite the fact that molecular machines based on the units of the above building blocks have come a long way, however, in the absence of any cooperative behavior, the mode of operation of molecular machines is still confined to the nanoscale. This solitary mode of operation not only limits the efficiency and complexity of the tasks that molecular machines can perform, but also makes it difficult to use their mechanically relevant motions to produce macroscopically effective work, especially in isotropic liquids [2, 115]. In order to solve the above problems, molecular machines amplify and aggregate the original individual behaviors into collective behaviors through collective behaviors. Two typical collective models have been widely studied and reported. In this section, their construction strategies and points of concern are discussed.

### Macroscopic Work Produced by Amplification

**Fig. 3:**
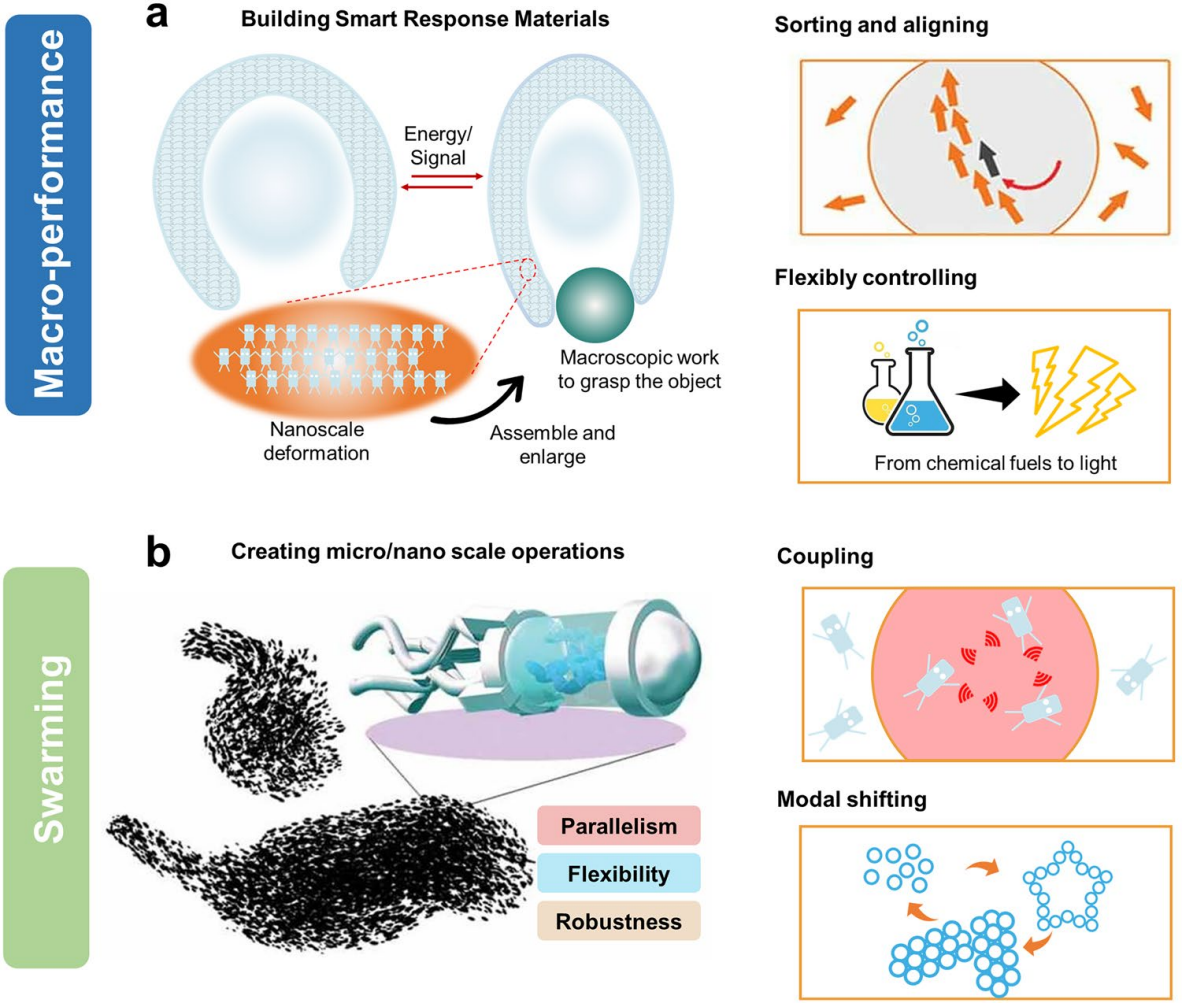
Collective behavioral patterns of molecular machines. **a** Molecular machines grasp objects by self-assembling or integrating into polymer networks that amplify nanometer mechanical motion to produce macroscopic work. This is expected to be used to build smart responsive materials. Two key issues in the design process are aligning and flexible controlling. **b** Molecular machines operate at micro/nanoscale by swarming. Swarming has parallelism, flexibility and robustness compared to individual behavior. During the design process, the focus needs to consider coupling and mode shifting. Reprinted permission from Ref. [90]. Copyright 2020, Taylor&Francis

In muscle tissue, it is elegant to amplify the motions generated by individual molecular motors into macroscopic motions. Large amounts of actin bind through myofilaments to form myofibrils that produce sarcomeres contraction and diastole. An advantage of molecular machines is that the presence of a large number of surface groups allows them to self-assemble into networks or integrate into hierarchical components such as crystals, liquid crystals (LC), and gels [115, 116]. Through this collective behavior, relatively weak and simple individual molecular machines conduct nano functions generated by conformational shifts through the connections of the network to produce macroscopically effective work. This opens up the possibility of building smart materials. The advantages of such materials are clear. They have controllable responsiveness to light or heat. More promisingly, the ordered arrangement of molecular machines, especially the incorporation of chiral molecules, can bring anisotropy to the materials, which will facilitate the development of soft robots (Fig. 3a).

In scaling up to macroscopic functionality, it is desired for materials to be able to have richer and controllable shape shifts. In order to achieve this goal, focusing into the collective behavior, the arrangement and alignment of molecular machines needs to be considered first. Molecular machines need to work together in space and time [117]. The variation of molecular machines at the nanoscale can only be maximized if they are in the same orientation to achieve an increase in energy conversion efficiency. Alignment also allows for better material anisotropy, which allows for the desired deformation in a specific direction. Controlling the orientation, especially of a large number of molecular machines, is quite difficult as the molecules are greatly affected by Brownian motion. This can be done on the one hand by designing the structure of the molecular machine in such a way that, for example, long strips of molecules tend to be ordered and aligned. On the other hand, it can also be achieved by active external control fields. Magnetic fields are advantageous in terms of alignment, but it is important to consider how to introduce the response units. Moreover, in some cases of successful amplification, it is often achieved by exploiting the ordered orientation of the network, e.g., LC. This strategy is ingenious. Effective controlled deformations are indeed realized, but they are usually one-dimensional. And expanding such one-dimensional transformations to two or even three dimensions requires molecular machines that differ in different orientations. Achieving this may still have to be combined with effective alignment of the controlling molecular machines.

The second key issue in implementing collective behavior is how to be more proactive in controlling it. The basis for molecular machines to achieve conformational changes lies in the need for energy from external sources to break the state of thermal equilibrium and achieve unidirectional motion. Despite the waste problem, chemical fuels have proved to be an attractive source of energy because they are readily available based on a variety of reversible chemical reactions, of which acid–base reactions are the most common [118–121]. Chemical drives, on the other hand, tend to be limited in their flexibility and require a more active source of control. Light, as a programmable physical field, enables controlled changes in time and space. This is crucial for the flexibility of macroscopic materials. In addition, light-driven motion allows us to bypass the principle of microscopic reversibility [115]. In photochemical reactions, large energy inputs allow reactions to take place on high-potential energy surfaces in the photoexcited state, where the molecules have a much higher degree of freedom to undergo transformations that are not permitted in chemically-driven systems (conformational changes, dissociation, aggregation, etc.) [122]. More importantly, photodynamic molecular machines lend themselves to autonomous operation [123]. This fits better with the design concept of smart materials.

### Swarming of Molecular Machines

At the supramolecular scale, the swarming behavioral patterns of molecular machines are expected to improve the ability to accomplish complex molecular tasks at the nanoscale. Swarming behaviors are exhibited in organisms such as birds, fish, cells and bacteria. These are effective in nature in helping organisms avoid and protect themselves from natural enemies, increase the likelihood of foraging, and promote reproduction. Patterns of integration and control of molecular machinery through interactions make collectives more efficient, flexible, and robust. First, the self-organizing nature of collective behavior allows relatively weak and simple individuals to perform more difficult and complex tasks. Second, the flexibility of the collectives maintains the adaptability of the system, and the flexible shape changes ensure that the molecular machine collectives can adapt to more complex environments. Finally, the robustness of the collective ensures that it will not collapse in a single blow, and even if some individuals break away from the collective, this will not significantly affect the nature and function of the whole. The very small size of molecular machines is a huge advantage in being able to create large numbers of machines and manage them to perform tasks in a consistent manner. Swarming of molecular machines would enrich and complicate what would otherwise be nanoscale micromanipulation (Fig. 3b).

Two important issues need to be considered during the execution of swarming motion. The first question is, how can interaction and coupling between molecular machines be achieved? The most important feature that distinguishes collective behavior from individual behavior is that it involves coupling and cooperation between collectives [124]. It is as if organisms can process information gained by interacting with nearby individuals and cooperate to change their organization, such as the location, shape or size of the collective, even in the absence of any leader. Thus, achieving interaction and coupling between molecular machines requires that they not be haphazard, but organically integrated. Specificity is a vital indicator of coupling to achieve correct matching, binding and disconnection, which is the basis for precise control of collective behavior and the guarantee of intelligence. In addition, the strength of coupling between individuals directly determines the speed of signal transmission between collectives as well as the intelligence of cluster behavior. Weak coupling will not be sufficient to integrate and amplify the small forces of individuals, and will lead to the loss of signals in the transmission process. Extremely strong coupling, on the other hand, will limit the process of dispersal, thus creating an obstacle to the intelligence of the collective behavior.

The second key issue in the process of implementing collective behavior is how to achieve an effective shift in collective modality. In this problem, flexibility is considered first. Flexibility tests the programmability of the control field, so external physical fields remain the primary consideration. With the exception of light, magnetic fields have more flexible programmability. Field strength, frequency, and phase are all tunable variables, and coil magnetic fields can be assembled into three dimensions. However, it is currently rarely applied to the control of molecular machines because magnetic fields are difficult to convert into energy for the molecules themselves. However, magnetic fields are not impossible, as they can also indirectly control molecular machines by altering their surroundings. This requires further design of the structure of the molecular machines themselves, as well as consideration of their interaction with the environment. Secondly, consideration needs to be given to how modularity can be constructed and orthogonally controlled. Realizing multimodality on a collective requires achieving modular integration rather than structural integration. Because, realizing multifunctionality on one kind of collective will greatly increase the complexity of the design. An effective strategy is to build heterogeneous collective through modularization, like compartmentalization within a cell. The multi-module design is more testing of control orthogonality, i.e., controlling one collective while having no effect on another. This not only requires the control field to effectively control all molecular machines, but also ensures the specificity of the control signals at different locations at different time points. This is crucial for building heterogeneous ensembles of modular molecular machine clusters to construct cell-like ordered smart factories.

## Feature Regulation of Collective Behavior

The design strategies in Sect. 3 are reflected in applied work based on two typical collective models. In this section, recent advances in both types of collective behavior in molecular machines are reviewed, and how these strategies are reflected in the design of specific collective structures and the modulation of features is discussed.

### Responsive Smart Material Construction

The bending, twisting and jumping motions of the molecular machines are added to other networks to be integrated and amplified. The molecular machines act as key joints responding to incoming signals to produce conformational changes and, through the transmission of the network, pass this change on to neighboring individuals to produce macroscopic changes that are sufficiently pronounced. In this section, the focus will be summarized in terms of reversibility, amplification, anisotropy, and reconfigurability.

Feringa group pioneered the development of a low molecular weight gelator (LMWG) [125]. This molecular machine is capable of undergoing *trans* to *cis* isomerization in response to ultraviolet (UV) irradiation, thus triggering a gel-sol phase transition. The process can be reversed by light irradiation and the gel phase regained later. Building on the inspiration of the work of Feringa group, Giuseppone group constructed chemically crosslinked polymer-motor couplings [126]. The unidirectional rotation of molecular motors fueled by light and working out of equilibrium are able to co-operate in a fully integrated sub-stable system. By twisting the polymer chains, powerful macroscopic contractions can be generated. Undoubtedly, this pioneering work not only provides indirection for the smooth incorporation of rotary motors into the gel network and the conversion of torque into radial force. It also answers many of the factors that need to be considered, such as whether motors degrade when exposed to light for long periods of time under mechanical constraints and whether rotational performance is affected by the length of the polymer chains. In addition to inducing macroscopic work, this material is also capable of storing energy from light. However, unidirectional rotation introduces irreversible shrinkage that can limit its application.

**Fig. 4:**
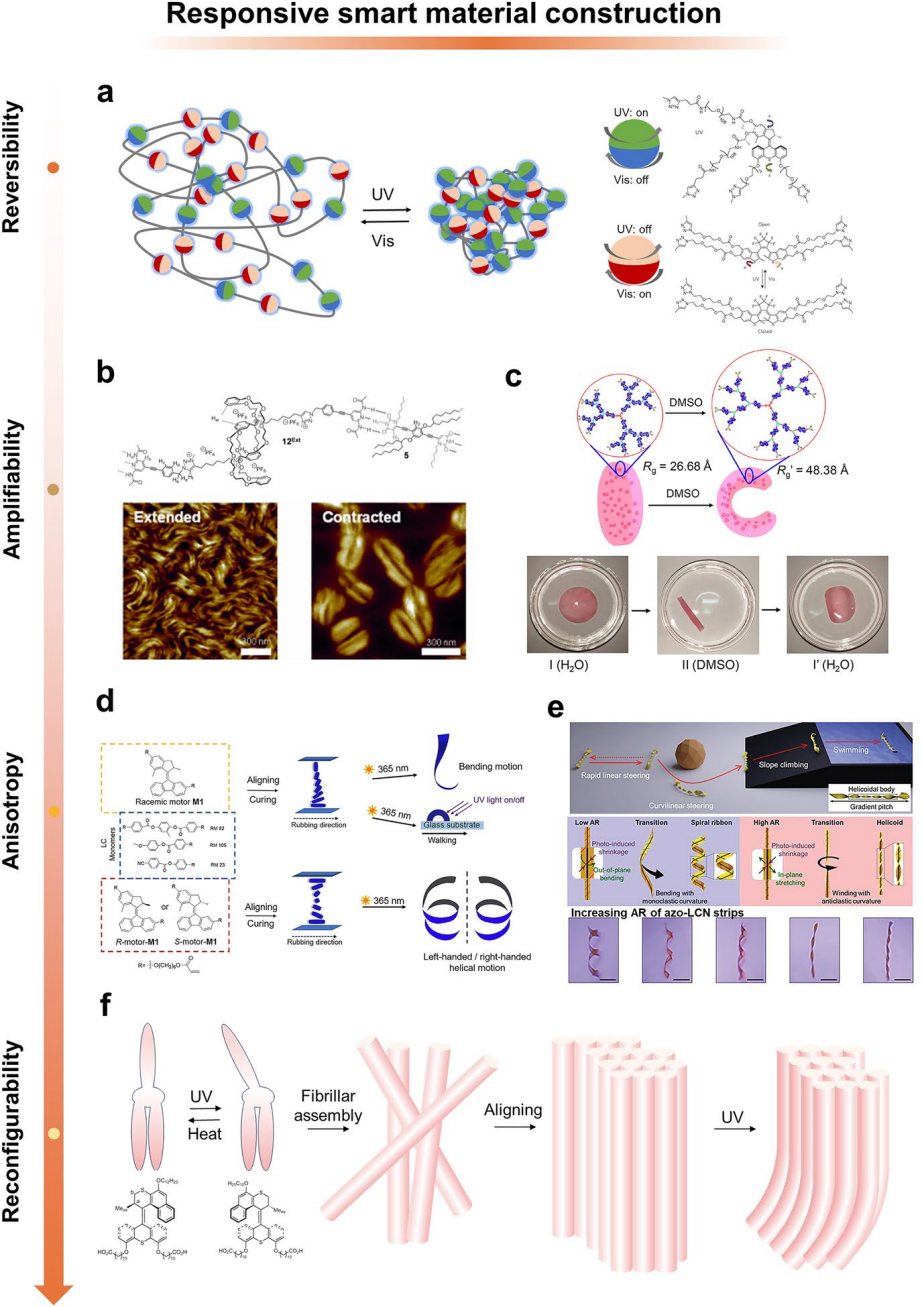
Responsive smart material construction. **a** A polymer with dual optical control based on integrated motors and modulators. UV irradiation induces motor rotation of polymer chain entanglement. VIS light is then used to activate the optical switch to produce its open form. Free rotation effectively releases the elasticity accumulated during motor rotation. **b** Molecular machines with single chains arranged in bundles to form a layered structure, like myofibrils. Reprinted permission from Ref. [128]. Copyright 2016, Wiley–VCH. **c** Third-generation daisy-chained dendritic polymers with 21 [c2] daisy-chain-rotor alkane portions. Collective and amplified extension/contraction through each [c2] chrysanthemum-chain-wheel-oxidized alkane branch upon addition of acetate anions or dimethyl sulfoxide (DMSO) molecules as external stimuli. Reprinted permission from Ref. [129]. Copyright 2020, American Chemical Society. **d** Monomers (blue dashed square) with racemic motor (R,S)-M1 (yellow dashed square). A mixture of the LC monomers and racemic motor (R,S)-M1 is aligned from homeotropic to planar. The mixture is cured into a homogeneous film and is cut along the rubbing direction. The obtained ribbon is able to bend upon UV light irradiation (365 nm) or walk over a surface. Monomers with enantiomerically pure motors (R)-M1 and (S)-M1 (red dashed square). The monomers are mixed with (R)-M1 or (S)-M1 motor and aligned into a twisted nematic structure. The mixture is cured into a homogeneous film and is cut along the rubbing direction. The resulting ribbon with R-motor shows left-handed helical motion when irradiated with UV light, while the ribbon with S-motor shows right-handed helical motion. Reprinted permission from Ref. [130]. Copyright 2020, Wiley–VCH. **e** Schematic of photo-steerable rolling locomotors controllable on various terrains (top). Photo-structured winding process of spiral ribbon and helicoid helix structures according to the aspect ratio (AR) of the azobenzene-functionalized liquid crystal polymer networks (azo-LCN) strips (middle). Helix structures and their continuous buckling transition according to the AR of the azo-LCN strips(bottom). Reprinted permission from Ref. [131]. Copyright 2023, Wiley–VCH. **f** Structure of an optically responsive rotary motor and its self-assembly into nanofibers

To address this problem, the team reported another dual-light-controlled polymer based on integrated motors and modulators (Fig. 4a) [127]. The process of working with this system consists of two stages. First, UV irradiation induces motor rotation of the polymer chain entanglement similar to the previous work. When irradiation stops, the motor stops rotating and the material remains contracted. A photo switch is then activated with visible (VIS) light to produce its open form, which is free to rotate around the C–C bond. Free rotation effectively releases the elasticity accumulated during motor rotation until the system reaches thermodynamic equilibrium.

To integrate and amplify collective behaviors more efficiently, molecules require organized and ordered orientations to perform synergistic and amplification operations [132]. In this regard, translational molecular motors are more advantageous due to their elongated structure. An effective idea is to arrange the single-chain polymers of the molecular machine into bundles of layered structures to efficiently align the daisy chains in the direction of extension, as is done when myofibrillar fibers are packaged transversely in bundles of muscle fibers [133–136]. The Giuseppone group pioneered this concept (Fig. 4b) [128]. The [c2] daisy-chain rotamer uses two diacetyl aminopyridine units as plugs, as well as a complementary double-site connector containing two 1-hexyluracil portions at its ends. The double-site joint is modified with a branched alkyl chain to ensure solubility in organic solvents and to provide additional van der Waals interactions to stabilize the primary hydrogen bonding pattern in the concerted self-assembly. The mismatch between the polar properties of rotane unit (presence of crown ethers and ion pairs) and the nonpolar properties of junction would facilitate microphase separation-driven lateral aggregation of single-chain supramolecular polymers, thereby enhancing the synergistic mechanism of supramolecular polymerization. In another work, chains can also be efficiently aligned into a typical hierarchical structure by individually immobilizing the chain ends on gold nanoparticles [137]. The chain is also efficiently aligned into a typical hierarchical structure by immobilizing the chains on gold nanoparticles. In addition, this work in reversibly controlling the proximity of the attached nano-objects, which is also instructive for micrometer-scale manipulation.

Another line of thought is in the construction of branched polymers by building branched polymers in order to assemble more molecular machine joints or even to form dendritic structures. For example, the reported crosslinked topology of branched polymerization of [c3] daisy-chain rotaxanes can contract and expand by changing the protonation state of the system by ∼50% of the volume [138]. No longer limited to one-dimensional biological muscles, this mode of assembly is capable of contraction and stretching in space [139]. Yang group reported daisy-chain dendritic polymers, which may be one of the most complex discrete higher-order mechanically interlocking molecules consisting of multiple [c2] daisy-chain rotaxane units (Fig. 4c) [129]. The daisy-chained dendritic polymers were endowed with controllable and reversible nanoscale size tuning by the addition of acetate anions or DMSO molecules as external stimuli. Recent studies have shown that the ability of daisy chains to produce significant macroscopic changes is due not only to the integration of sliding by molecular machines in the network, but also to the effective modulation of stress distributions on conventional polymer chains. This was demonstrated in the work of Yu group [140]. They endowed the molecular machine network with good tensile, toughness and damping capabilities by introducing primitive dangling chains into the network. In addition, they found that rotaxanes, as key functional units, can achieve significant macroscopic effects by modulating the stresses even when the system has a relatively low content of rotaxane units. This provides a new understanding of the collective mode of this class of molecular machines. Outside of the traditional regulation dominated by pH and ion concentration modulation, the collective and amplified motion of precisely aligned rotaxane units, resulting from the introduction of photoisomerization of the azo units, leads to controlled and reversible size modulation of photo responsive rotaxane-branched dendritic polymers integrated in solution [141].

In the above behavior of contraction and expansion, there is only a change along the radial direction. It is difficult to form an effective functionality for such transformations. Thus, the realization of macroscopic anisotropic shape shifts is a manifestation of the intelligibility of this collective paradigm. The combination of LC and molecular machines is a paradigm often used in this work. LC exhibit highly ordered structures with anisotropy induced in solution or in the molten state, providing a favorable collective environment for molecular machines. The long-range orientation order of LC also enhances the effectiveness of chiral transfer from the molecular scale upwards [142]. The molecular machinery can be aligned with molecules forming nematic or cholesteric LC, whose shape changes can be induced by inducing anisotropic shape shifts by inducing disorder and applying molecular forces to the polymer network [115]. Azobenzene open light is most commonly used to activate the liquid crystal network(LCN) [143]. Azobenzene is able to achieve reversible transformations between rod (*trans*) and crescent (*cis*) forms after cyclic irradiation with VIS and UV light. In 2004, Peter's team doped azobenzene into a LCN that produces mechanical deformations of more than 60° in response to inhomogeneous illumination in the VIS light [144]. Another molecular machine with carbon–carbon double bonds as the axis of rotation produces, when illuminated under UV light, a unidirectional rotation [145]. Doping at only 1% mass ratio was sufficient to display a fingerprint texture on the LC film, which, by rotating the texture, was able to produce feats of rotating sub-millimeter size particles. By further doping with chiral molecules, molecular machines are able to induce macroscopic oriented helical structures [130, 131, 146]. Feringa has realized complex mechanical motions, including bending, walking, and helical motions, in soft polymer materials by exploiting the dynamic chirality, motion directionality, and shape variation of individual motors embedded in LCN (Fig. 4d) [130]. In this responsive system, the photochemically driven molecular motors have a dual function of acting as chiral dopants and operating as unidirectional rotors that amplify the molecular motions into controlled and reversible left- or right-handed macroscopic torsional motions. In this type of work, light actuation is advantageous because lighting conditions can be spatially organized, which is more beneficial at the level of control. For example, through inhomogeneous illumination, it is possible to induce inhomogeneous phase transition expansions, resulting in the formation of miniature swimmers with traveling wave motion [147]. In another work, macroscopic light-guided bending deformations along the helical axis can be stabilized to arbitrarily control the rolling direction when patterned light is irradiated onto a central tapered helical structure (Fig. 4e) [131]. At a higher level of demand, autonomy is desired. This is exemplified in the work of Selinger's team. They further improved the *cis–trans* isomerization conversion of azobenzene by adding push–pull groups and forming azobenzene that can be isomerized with each other. This allowed for a rapid transition of the molecular machinery when moving from light into shadow. This resulted in polymer films that exhibit continuous mechanical waves in constant light with feedback loops driven by shadows [148].

Reconfigurability is a key indicator of whether collective behavior can achieve multidimensional transformation. In order to overcome the limitations of strong coupling and cross-linking on the degree of freedom of collective behavior, the idea of using molecular machines to synergize into supramolecular assemblies has been proposed. Feringa and colleagues report a work that relies on non-covalent interactions of light-responsive non-polymer-based molecules can maintain muscle-like movements in water (Fig. 4f) [149]. These light-driven molecular motors have a central double bond around which they undergo unidirectional rotation. The rotation is encoded as unidirectional by setting up a spatial site resistance. Amphiphilic molecular motors on multiple length scales were organized into long supramolecular polymers to form nanofibers with a diameter of ∼5–6 nm. When these nanofibers were added to an aqueous CaCl_2_ solution, forces between carboxylates and calcium cations coupled the fiber bundles, self-organizing them into bundles with unidirectional alignment. Under light irradiation, light-induced rotation of the motors in the fiber bundles initiated the contraction of the nanofibers along the long axis while their diameters expanded to maintain the total volume of the isovolumetric shape transformation. Relaxation of the motors to the original molecular conformation is facilitated by thermal helical inversion at the level of assembly, which corresponds to the material recovering its original shape. This mode of assembly would provide additional benefits for future biologically relevant applications due to the highly dynamic nature of the material (ready-to-assemble-disassembly), although collaborative molecular motions are usually hindered by the instability of the supramolecular structure during the molecular shape transformation process, which results in poor mechanical properties of the supramolecular assembly. This work provides encouraging evidence for the self-assembly of molecular machines, but such morphing materials are essentially still one-dimensional transformations. Achieving collective behavior in higher dimensions will require consideration of not only the design of dynamic molecular components, but also their coupling to their functional environment.

In addition to these major collective models, there have been some new integration ideas recently. For example, the artificial reconstruction of muscles with motor proteins is highly anticipated [150–155]. A collagen-actin (CA) hybrid soft actuator inspired by the structure of natural muscle, called CA actuator, has been reported [156]. In CA actuators, polymerized actin filaments and myosin filaments are entangled with a collagen gel network. The actin contracts the entire CA actuator by bringing ATP together with the entangled collagen gel network. In addition, supramolecular self-assembly materials with tannic acid as the building block can bind proteins, and have great medical application potential in drug delivery, tumor diagnosis, and therapy [157]. Recently, work on incorporating molecular machines into 3D solid materials has also been reported. Feringa successfully introduced molecular motors into a crystalline metal–organic framework (MOF) [158]. Based on the MOF platform, light-driven rotating molecular motors can be organized into a solid state to achieve a well-defined spatial organization. Detailed studies show that such solid-state molecular motors perform complete rotational cycles and achieve similar rotational speeds as in solution. These "moto-MOFs" open a new door for dynamically tuning MOF behavior.

Effective amplification of microscopic forces to macroscopic scales is achieved by orderly integration of molecular machines into other polymer networks. However, this morphing material is limited to bending, contraction and expansion, essentially a transformation that still has only one degree of freedom. Furthermore, although self-assembly and LC can act through weak cross-linking, most of the forces in the other modes are strong forces formed by covalent bonds. The ability of this collective behavior to be reconfigurable is greatly limited because the assembled molecular machine-polymer network is difficult to reconfigure. There is still a long way to go, both in terms of the complexity of the shape changes and the versatility of the resulting patterns of movement and operation [115].

### Swarming of Molecular Machines

Over the past decade, inspired by the swarming of bees, there has been a growing interest in the swarming of molecular machines, especially biomolecular machines. Unlike the last collective behavior, the scale of this mode is in nanometers or micrometers. This collective mode stems from weak interaction forces such as hydrogen bonding interactions or van der Waals forces between molecules and their interactions, rather than strong interaction forces based on covalent bonds predominantly. As a result, the collective behavior is more flexible and reconfigurable. Cargo handling, micromanipulation, etc. at the micro- and nanoscale are the desired application sites for this collective model. In this section, the focus is summarized in terms of collective formation, reconfigurability, orthogonality, and logical control.

**Fig. 5:**
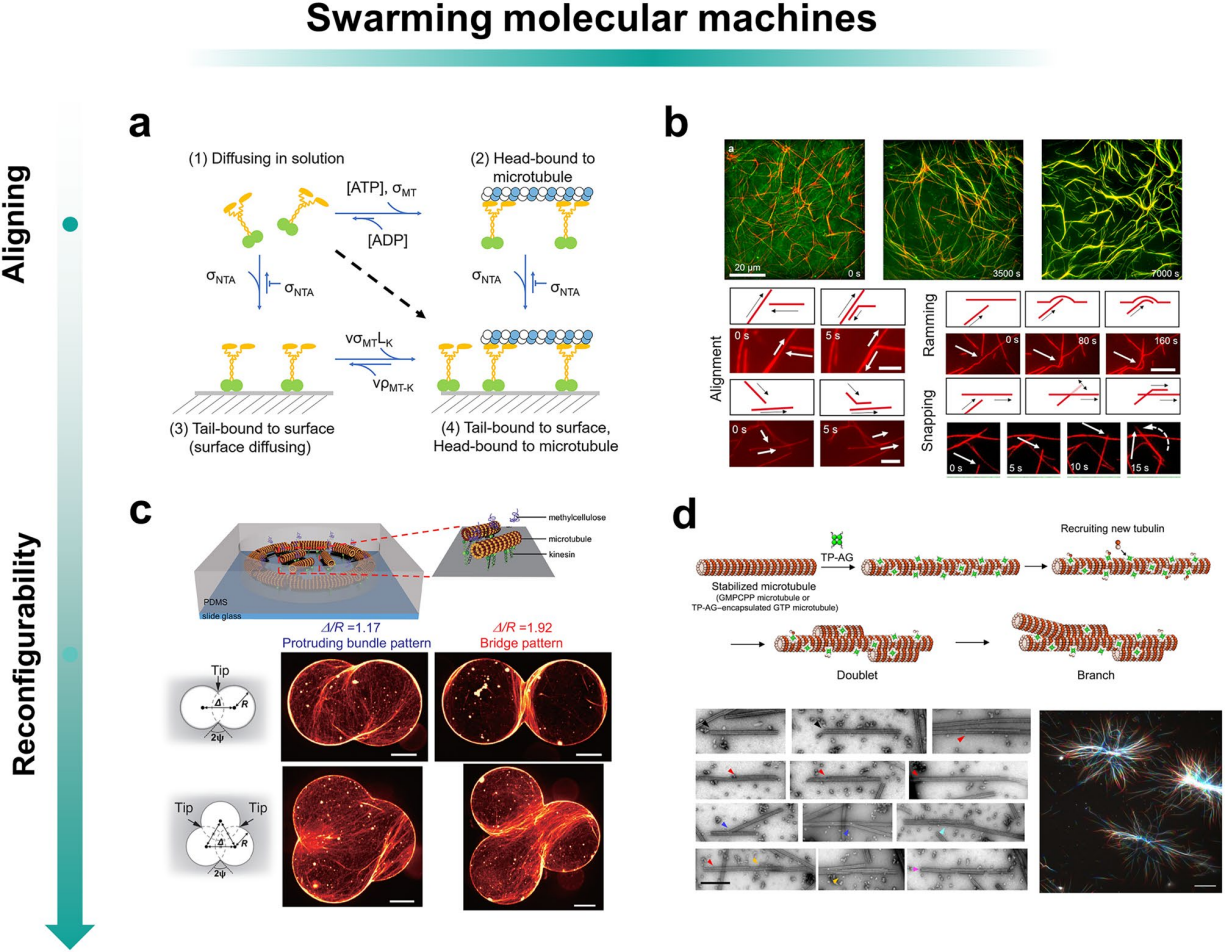
Swarming molecular machines. **a** Schematic diagram of motor binding states. In a dynamic system, the motor can enter four states: diffusion in solution, head-bound to microtubules, tail-bound to surface, head-bound to microtubules, and tail-bound to surface. **b** Microtubules assemble into bundles when green fluorescent protein (GFP)-kinesin aggregates on microtubules (top). Mechanisms of microtubule alignment (bottom). Reprinted permission from Ref. [159]. Copyright 2020, American Chemical Society. **c** MTs move in response to forces exerted by kinesin motor proteins (top). Assembly pattern of MTs enclosed in double-incorporated ring microwells during 60 mins (middle). At Δ/R = 1.17 (left), a protruding bundle pattern of MTs can be observed from the tip, whereas a bridging pattern of MT bundles can be observed at Δ/R = 1.92 (right). Assembly pattern of MTs confined in triple-conjugate circular microtiter wells within 60 mins (bottom). At Δ/R = 1.17, a similar protruding bundle-like pattern is formed from the tip. In contrast, these protrusions form bridges between the three tips at Δ/R = 1.92. Reprinted permission from Ref. [160]. Copyright 2021, American Chemical Society. **d** Models of bimodal and branching microtubules and the different morphologies of microtubules were formed with Tau-derived peptide-Azami-Green (TP-AG), single-linear state microtubules (black arrows), bimodal microtubules (red arrows), branching microtubules (blue arrows), multimodal microtubules (orange arrows), branching and multimodal microtubules (cyan arrowheads), and aster-like. Reprinted permission from Ref. [161]. Copyright 2022, AAAS

Kinesins and MTs are the classical system for analyzing how simple interactions between intelligences lead to dynamic self-assembly and collective behavior [162–171]. Hess group studied the dynamics of MT movement (Fig. 5a, b) [159]. They observed that the MT movement velocity decreased from an initial rate of 720 ± 20 to 16 ± 1 nm s^−1^ over a period of two hours. During this period, sliding MTs formed bundles, and kinesins from an initial uniform distribution to aggregation in the MTs. They noted that collision-induced nematic alignment is one of the main reasons for the formation of collective behavior. This is because collisions occur when two MTs are oriented differently. The weaker side will be forced to change its orientation until it aligns with the other side, at which point the collision will be avoided. The swarm alignment brought about by collisions not only allows vascular bundles to spontaneously organize themselves into millimeter-sized wires [169], but can also lead to the formation of large vortices [172]. In addition, crowded space is an important factor in the formation of vascular bundles because it contributes to the probability of collisions. In another work, the use of polyethylene glycol (PEG) to force dense stacking of MTs constructed assemblies capable of generating beating patterns similar to those found in eukaryotic cilia and flagella [173]. They self-assemble into active bundles and act on flexibility within the MT bundles with the energy supply of hundreds of molecular motors, and the alignment effect is capable of spontaneously synchronizing their beating patterns to produce a collective behavior of temporal waves. In addition to these two effects, the cross-linking of kinesins also contributes to vascular bundle formation. Kinesins bind tightly to the microscale and affect the dynamics of MT sliding [174]. In this dynamic system, MTs produce many attachments to the surface through dense kinesin motors recruited from solution. Dense surface attachment reduces vertical fluctuations and prevents MTs from crossing each other, forcing them to bend and align with the MTs they collide. Collective behavioral alignment and cross-linking interactions enable the spontaneous organization of vascular bundles into millimeter-sized wires [169], and collisional interactions lead to the formation of large vortices [172]. By regulating access to the appropriate kinesin-surface interaction forces, constructing mutually beneficial collective behaviors in a system with two mobile agents (kinesins and MTs), but also demonstrating the possibility of higher-order self-assembly of motors and bundles of tubules assembled hierarchically [159].

On the basis of understanding collective formation, choosing appropriate means to increase the reconfigurability and diversity of collective behavior becomes the next goal. Controlling the collective movement of kinesin-driven MTs through topographical patterning is a simple and ingenious way to do this. For example, creating closed chamber walls improves MT interactions. The number of end-to-end MT fusion events increases 20-fold compared to MTs sliding on a flat surface [175]. This is because the increase in spatial bit resistance raises the probability of collision. In the work of Shunya Araki, devices with double- and triple-circle boundaries were constructed to demonstrate geometrical control of self-propelled MTs (Fig. 5c) [160]. Different boundary shapes change the collision angle of sliding MTs, which can control the self-assembly of MTs to form prominent beam and bridge patterns. And further, the shape of bundles and bridges is controlled by changing the distance between two circles. Another way to modulate the collective behavior is by modifying MTs to change the coupling behavior between individuals. For example, the tetrameric fluorescent protein Azami-Green (AG) was used to fuse with a His tag and a Tau-derived peptide (TP) to produce supramolecular structures (Fig. 5d). By varying the polymerization conditions, the primary binding site of TP-AG to the inside and outside of the MT can be controlled. The binding of TP-AG to the inside promotes MT formation and produces rigid and stable MTs. In contrast, the binding of TP-AG to the exterior of MTs induced a variety of MT superstructures, including double-stranded bodies, multiplexes, branching structures, and ultra-long MTs, and also induced the generation of aster-like structures [161].

Another important class of biological macromolecules is DNA. DNA origami technology is capable of building complex nanostructures with multiple single strands. Thus DNA itself can act as a molecular machine, moving and sorting molecular cargoes across the surface of DNA origami [176]. However, work in DNA origami technology has focused primarily on building single molecular machines, and there has been very limited work on using DNA molecular machines in isolation to build collective behaviors. More valuable work has utilized DNA to modulate coupling relationships between MTs. DNA has high molecular recognition. Thus, when used for coupling between individuals, it ensures efficient specificity and opens new possibilities for reconfigurability and logical manipulation of collective behavior.

**Fig. 6:**
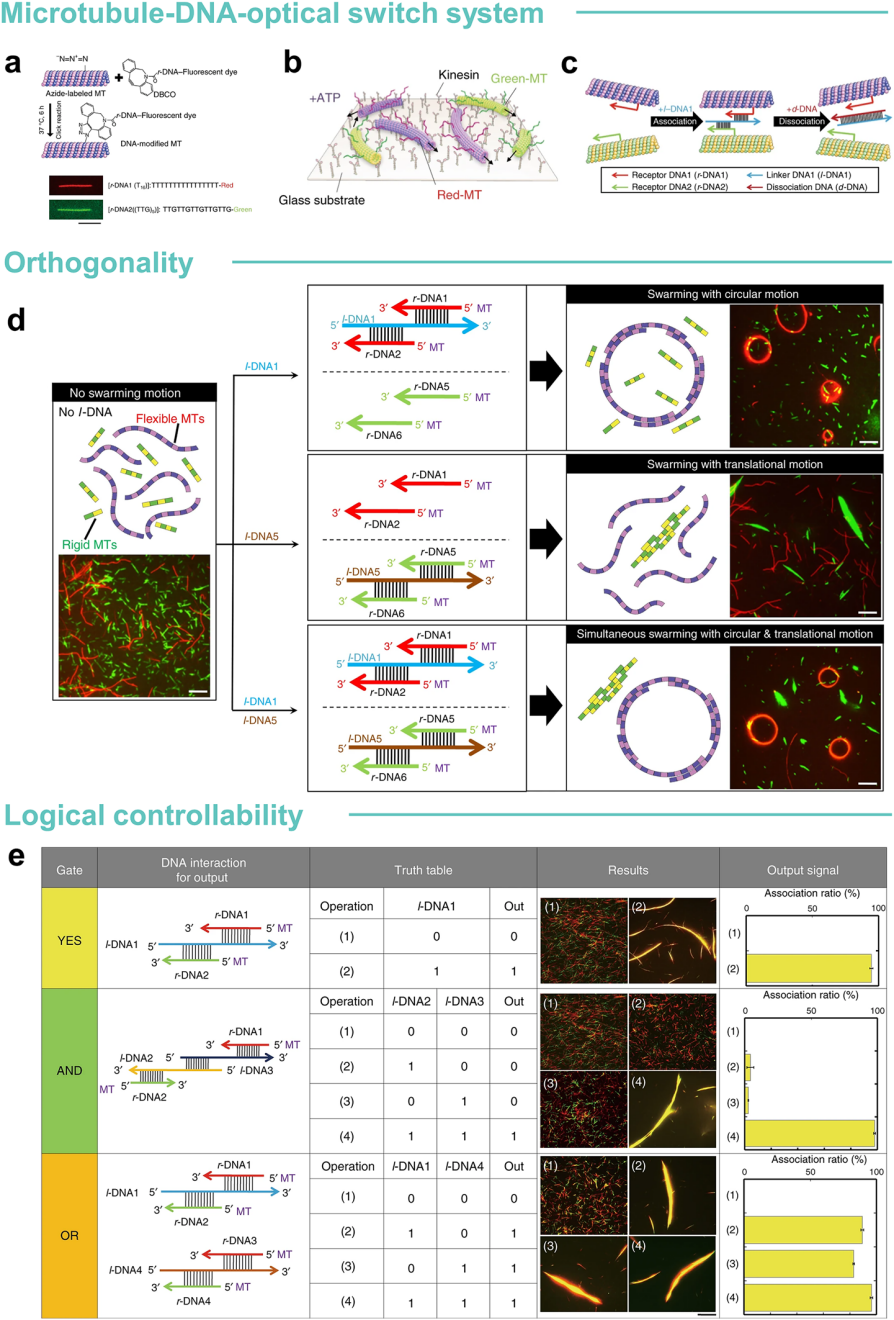
Microtubule-DNA-optical switch system. **a** Conjugation of *r*-DNA to azide-functionalized MTs by a click reaction. **b** Schematic of red and green MTs gliding on kinesins. **c** Schematic of the association of red and green MTs by *l*-DNA1 and their dissociation by *d*-DNA via extraction of *l*-DNA1 through a DNA strand exchange reaction. **d** Orthogonal control of swarming of MTs. The flexible MTs (red) were conjugated with *r*-DNA1 and *r*-DNA2, while the rigid MTs (green) were conjugated with *r*-DNA5 and *r*-DNA6. Upon inputting *l*-DNA1, the flexible MTs associated into circular shaped swarms through hybridization of *r*-DNA1 and *r*-DNA2 with *l*-DNA1 and appeared in red. Green swarms with translational motion associated with rigid MTs were formed through hybridization of *r*-DNA5 and *r*-DNA6 with *l*-DNA5. Swarms with translational and circular motions were simultaneously formed in response to the introduction of both input DNA signals. **e** Design of logic gates constructed with MTs. For the YES gate, the *l*-DNA1 signal was inputted into the system and swarming was obtained as the output signal (1 to 1). For the AND gate, *l*-DNA2 and *l*-DNA3 had both to be present to obtain swarming. For the OR gate, the presence of either *l*-DNA1 or *l*-DNA4 was sufficient to obtain swarming. Reprinted permission from Ref. [165]. Copyright 2018, Springer Nature

Molecular machines using azobenzene as a sensor, DNA as an information processor, and kinesins and MTs as actuators are the classic paradigm for intelligent collective control (Fig. 6a–c) [90]. In the work of both Mousumi Akter, azobenzene was doped into photoresponsive DNA (pDNA) and coupled with MTs to achieve photo controlled regulation of persistence of MT travelling paths and speed of movement [177]. MTs have single-stranded light-responsive DNA attached to them and frequently make contact and collide with other MTs around them in the presence of kinesin. Under VIS light, the azobenzene is in a *trans* structure, and the small spatial site resistance allows the DNA strand to polymerize easily with neighboring strands. On the contrary, under ultraviolet light, the *cis* structure of azobenzene creates a large spatial site resistance to the double strand, which leads to the dissociation of the double strand. With the same design principle in mind, the successful loading and movement of cargo to a designated location by a swarm, which would be insurmountable for a single transporter, was achieved [178].

This assemblage based on the construction of DNA molecules is applied to spatio-temporal control to improve the diversity of collective behavior. A micrometer-sized robotic collective of liposome molecules similar to amoebas is reported [179]. The presence of clutches is key to controlling whether the liposome deforms or not. A kinesin attached to a single-stranded DNA is able to bind to the MT, while the anchor of another segment of single-stranded DNA is fixed to the liposome membrane. Remarkably, these two segments of DNA are not complementary. The light-responsive DNA, which is complementary to each of the two DNA segments, was designed as a double-stranded hairpin structure, and the double strand was exposed upon light activation to connect the anchor to the kinesin. Ultimately, the rigidity of the MTs produces shape changes in the liposome membrane by moving them. This is a good example of the integration of components of a robot into a functional system. DNA origami technology, which enables the construction of complex nanostructures with multiple single strands, also opens up the possibility of rich modalities for collective behavior. Kuzuya group reported that DNA origami nanostructures mediate the self-assembly of MTs and kinesins. They fabricated a 6-helix bundle of tubular structures surrounded by a large number of single strands that induced multiple MTs to contract like smooth muscle, and aggregates to form an aster-like structure [180]. This work successfully demonstrated the programmed self-assembly of a biomolecular motor system.

Further improvement of the orthogonality of the control of molecular machines based on the classical paradigm is the key to move towards modularity and integration. Orthogonality has been one of the biggest challenges in molecular robotics operations, especially in terms of their swarming. Orthogonality in the context of swarm robotics is defined as a characteristic that enables different groups/types of robots to perform clustered or individual activities without interacting or interfering with each other [90]. Akira Kakugo's team targeted optical switches [181]. Previously reported examples have utilized a single optical switch alone, thus limiting further applications of swarming systems, such as the transport of different types of goods from one place to another or sorting multiple goods at the same time. They introduced a new photo switch in DNA, tert-butyl-substituted azobenzene, which allows ON and OFF switching of MT clustering in a manner opposite to the VIS and UV-induced MT clustering and dissociation of the azobenzene switch. This work will broaden the application of biomolecular motor-based clustering systems for goods sorting in different locations and improve orthogonal controllability. Differences in MTs also act as degrees of freedom for clustering behavior to improve orthogonality (Fig. 6d) [165]. The control of the swarm was accompanied by variable stiffness of the MTs, which successfully formed translational and circular motions; furthermore, the morphological changes of the swarm depended not only on the stiffness of the MTs but also on the body length [166]. The strength of DNA interactions was modulated by varying the concentrations of receptor DNA (*r*-DNA), linker DNA (*l*-DNA), and dissociation DNA (*d*-DNA), all of which were shown to have an effect on MT swarming [166]. In addition, another mechanochemically coupled reactive–diffusive active-substance system constructed by self-amplification of the DNA strand, the kinesin microsystem, was reported [182]. The mechanochemical coupling between the two subsystems was achieved by increasing the strength of the flow generated by the active gel, which induced four different MT structures and two DNA patterns.

DNA has the ability to store large amounts of data, promising to be used to build data processors for collective behavior. A system combining a biomolecular motor and DNA is reported [183]. The motors and DNA-based nanoarchitectures enabled us to arrange the binding sites on the track, locally control the direction of movement, and achieve multiplexed cargo transport by different motors. Using DNA's ability to act as a logic operator in molecular computation, molecular robots demonstrate different logical operations such as YES, AND, and OR logic-gates, which determine different behaviors of clusters of molecular robots (Fig. 6e) [165]. The YES logic-gate is implemented by using *l*-DNA1 as input and swarms of red and green MTs as output. The swarming behavior will be generated when the input signal is present. AND logic-gate is implemented by designing two different joint DNA signals as *l*-DNA2 and *l*-DNA3, which are partially complementary to *r*-DNA1 and *r*-DNA2, respectively, and to each other. Clusters of molecular robots were observed as outputs only when both input DNA signals were present. OR logic-gate was implemented by coupling *r*-DNA pairs to MT and using *l*-DNA1 (complementary to *r*-DNA1 and *r*-DNA2) and *l*-DNA4 (complementary to *r*-DNA3 and *r*-DNA4) as the two input signals. These two types of robots independently exhibited swarming. When both input DNA signals were available in the same swarm system, both swarms operated in a coordinated manner. The logical operations of such molecular robots based on DNA molecular computation are highly advantageous and have not yet been realized in other swarm robotic systems [90].

By assembling biomolecular motors, DNA, and photosensitive molecules, swarms of molecular robots have been executed as an emerging function. The addition of DNA gives molecular robots the ability to optimize the processing, storing, and transmitting of information, but more future advances are still needed. In addition, molecular robots or entirely new frameworks with more complex structures and functions, based on DNA origami techniques, are being incorporated in various combinations for collective control. It can be said that collective control of molecular machines based on DNA molecules has a bright future as a hot research area.

## Grand Challenges

**Fig. 7:**
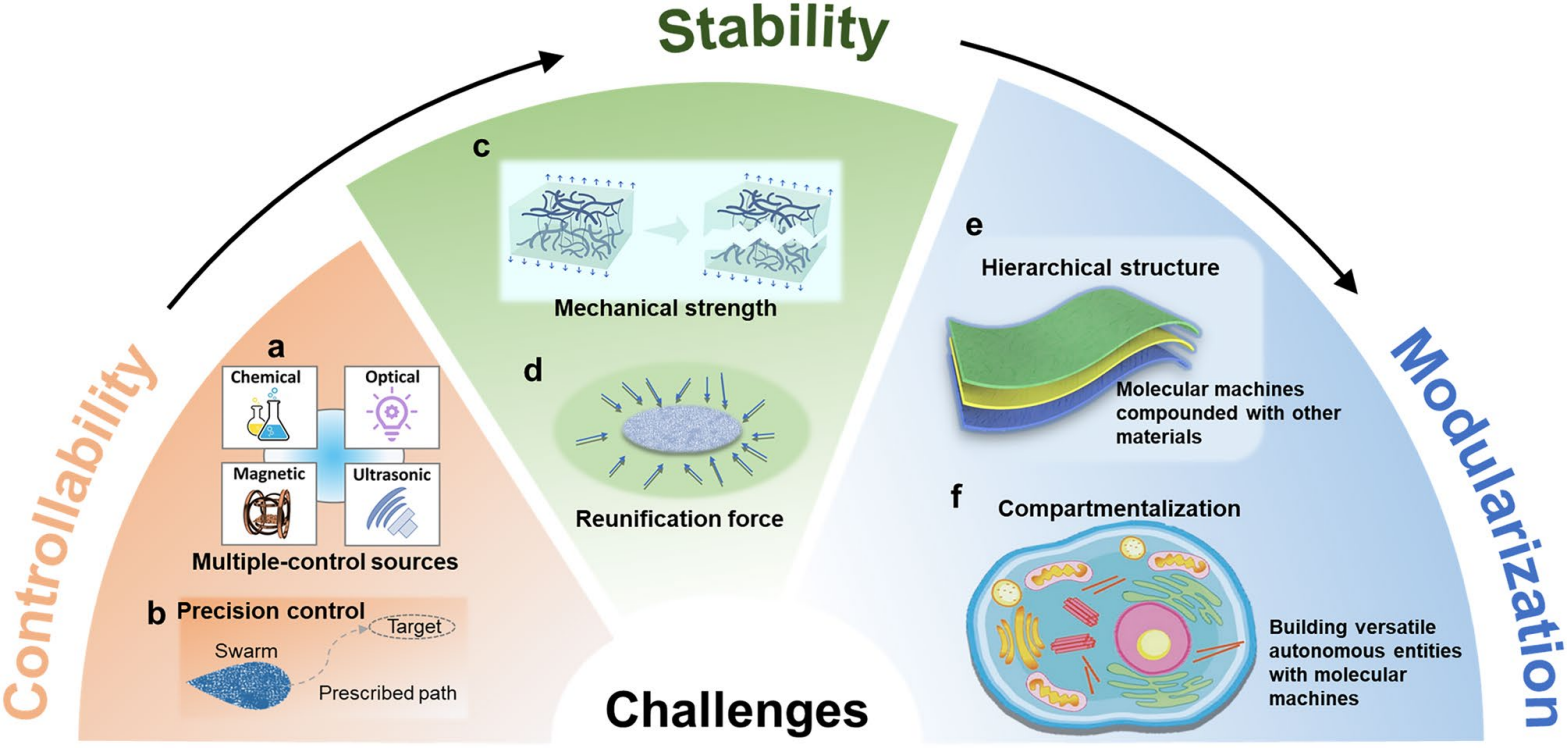
Challenges in the collective behavior of molecular machines, from controllability and stability to multimodularity. **a** Integration of multiple control sources may increase the dimensionality of modal shifts. **b** Swarm needs to be precisely controlled, including modes, paths, and targets. **c** The collective needs to ensure sufficient mechanical strength to prevent internal fracture during mission execution. **d** Specific forces need to be in place to maintain the swarm's aggregation. **e** Smart materials need to be combined with other materials to form layered structures to be application-oriented. **f** The collective development goal is to form orderly modularity and integration, like cells. Reprinted permission from Ref. [184]. Copyright 2020, Wiley–VCH

The emergence of swarming behavior and its application to molecular machines has broadened the scale of application of molecular machines, which is getting closer to Prof. J. Fraser Stoddart's vision of molecular machines at all length scales. However, neither the use of molecular machines to build smart responsive materials nor the use of swarming behavior to perform nanoscale operations is perfect at this stage. Many of these models are at the conceptualization stage, which is far from the ideal application. It is necessary to revisit these two types of collective behaviors from the perspective of collective control, even though they have many differences in terms of application sites and construction strategies. Therefore, in this section, the focus is on summarizing the problems and challenges that are common to both types of collective behaviors in order to design better strategies to overcome them (Fig. 7).

### Multi-Dimensional Precision Control

The control dimension is key in determining the dimension of collective behavior. Both the deformation dimension of the material and the finite modes of the swarm are somewhat limited by the finite control dimension. For the most part, the collective behavior of molecular machines cannot be separated from light. This is reflected in the driving of molecular machines and in the regulation of collective behavior. As we discussed earlier, light has many advantages. However, the advantages of the spatio-temporal programmability of light have not been effectively exploited. This is limited by the structural design of molecular machines, and more ingenious strategies are needed to better utilize the advantages of light. Furthermore, the extension of similar integration principles to molecular motors fueled by chemical energy sources may be of great interest. The combination of control sources will inevitably increase the dimensionality of the control. This means that chemical switches would need to be effectively integrated with optical switches to create richer collective behavior. One even crazier vision is to incorporate other physical fields, such as magnetic, ultrasonic, and electric fields, into the realm of molecular machines, since all of these physical fields are also programmable for spatiotemporal control. The realization of this idea is difficult because molecular machines are all they cannot all produce responsiveness. But it must be affirmed that the inclusion of any of them is bound to bring new breakthroughs in molecular machines.

In addition to the control dimension, achieving accuracy is another important aspect of collective control applications of molecular machines. Imprecision in any dimension limits the practical applications of molecular machines. Currently, control strategies have been proposed to switch between the direction and degree of deformation of the material, as well as the speed of motion of the MTs and the behavior of different clusters. How does precision go about ensuring this? Cluster control is not like monolithic control, which needs to control the behavior of a large number of individuals. In the case of smart materials, inhomogeneities in the distribution of the molecular machinery lead to inhomogeneities in the deformation. The problem this poses is in the precision of the control. In the case of microtubes, their direction of motion depends on the random force between the drive motor and the microtubes, so there is no exact direction. This directly affects the realization of practical applications, such as the delivery of goods. This is because we do not want the microtubes to be able to simply push the goods, but to be able to deliver them to the correct position, i.e. addressable [176].

Further, the higher stage of control for molecular machines is that they have feedback loops. This means that they are able to adapt to their environment to produce continuous and autonomous execution. This embodies intelligence and automation. The interactive materials of the future must sense, respond to external stimuli, act autonomously and be self-regulating, and must process information at all levels. Considering molecular materials and molecular processes as information-rich information processing systems may provide new dimensions for material designers. Artificial intelligence methods and computational approaches may guide molecular information explorers. In this model, materials are both machines that perform tasks rhythmically and physical objects that perform tasks, just like living matter [115].

### Sufficient Stability

When faced with practical applications, an issue worth considering is the stability of the integrated components of the molecular machine. This relates to the ability of a molecular machine to successfully perform its intended task and recover intact. In many cases, stability is in contradiction with variability. For molecular machines, the larger the distance traveled, the larger the macroscopic deformation will be obtained, but at the same time, this brings about stability issues such as mechanical strength. Coupling individuals through strong covalent bonds ensures sufficient mechanical strength and stability, but this can limit the transformation between different behaviors. The main advantage of supramolecular self-assembling materials, which typically couple individuals via noncovalent bonds, may be their tendency to adapt, reconfigure, and change function. However, the ability of such combinations to maintain sufficient mechanical strength is a question worth considering. In the future, adjusting the strength of coupled interactions between individuals to achieve appropriate stability is a priority for applications.

For swarming behavior, not only the stability of the cluster needs to be considered, but equally important is the stability of the dispersed state. The dispersed state often appears in the switch between two different aggregation modes. In addition, the dispersed state can be used as a separate mode of operation to increase the coverage area for performing tasks. The stability of the dispersion process ensures that the molecular machinery can be smoothly reassembled into a collective rather than permanently separated from each other due to lack of mutual attraction. In DNA, MT and kinesin systems, DNA double-stranded polymerization between MTs is still achieved primarily through collisions of MTs through random motions. This requires the creation of artificially crowded environments in which they can approach without difficulty. In practice, however, one is faced with the possibility of being in fluid flow and cluttered environments, which will greatly test the stability of the molecular mechanism of dispersion. Strategies are needed to ensure that the dispersed state still has some force to maintain the distance between individuals. Where this force comes from and how to get the stability is a question worth pondering.

### Modularity and Integration

In living systems, each molecular machine is combined in a highly rational manner to achieve high functionality. On both existing collective models, the modules are singular. This is easy to achieve on macroscopic materials because we are able to manipulate them directly. However, no similar work has been reported. Responsive materials need to form layered structures with other components to form life-like materials, just as muscles, bones, skin, etc. together form an arm. The idea of modularity and integration is even more enticing in swarm behavior. Research on molecular machines nowadays considers the construction of the three most basic components: sensors, processors and actuators. For practical tasks, further functionalization is still needed to integrate other modules such as imaging and catalysis. Simple modules can be directly integrated into the structural design of molecular machines, e.g., the use of fluorescent dyes for labeling DNA and MTs [165].

However, for the integration of more modules, the focus should be shifted to functional synthesis rather than structural synthesis. This means that in order to realize a hierarchical structure, it is necessary to build in heterogeneous collective in molecular machines. Different modules have different collective functions. This will facilitate the integration into large collective systems and the construction of a multifunctional autonomous entity, i.e., a multitasking macroscopic machine composed of molecular machines. How to coordinate the multiple attributes of assembly, sensing, and response behavior is worthy of further consideration, as there is a need to ensure the robustness and responsiveness of each module to external interactions, signals, or triggers (e.g., interfaces, redox, light, pH, and temperature) [185]. In addition, it is now time to explore the programming aspects of controlling sequential tasks consisting of collective movements. The programmability of DNA opens up the possibility of solving this problem, but further design and validation is needed.

## Experience from Micro/Nanorobots

Micro/nanorobots are also a hot area at the forefront of science. They are often constructed from active nanoparticles or active microparticles, on which functional molecules and polymers are modified. There are many similarities between micro/nanorobots and molecular machines, e.g., they are both at the micrometer and nanometer scales and are desired for micro- and nanoscale operations, and they both have receptors to receive external stimuli and actuators to carry out motion and work. Early micro/nano-robots were dominated by inorganic metal compound materials, but as organic polymer materials and biomaterials continue to be added to the mix, micro/nanorobots are becoming closer to molecular machines. Collective behavior has been studied more maturely in microrobots than in molecular machines, and there have been many preliminary applications in the treatment of environmental pollutants [186–191], targeted drug/goods delivery [192–197], and in vivo imaging [198–202]. And, exhibiting extremely rich behaviors, such as 1D gathering and dispersing behavior, 2D multimodal pattern reconfiguration, 3D spatial assemblies, and 4D multifunctional hierarchical structures [203]. This exhibits extremely appealing charms [124]. Therefore, it is hoped that experience in the field of micro/nanorobotics can be utilized to guide molecular machines in order to break through the challenges.

**Fig. 8:**
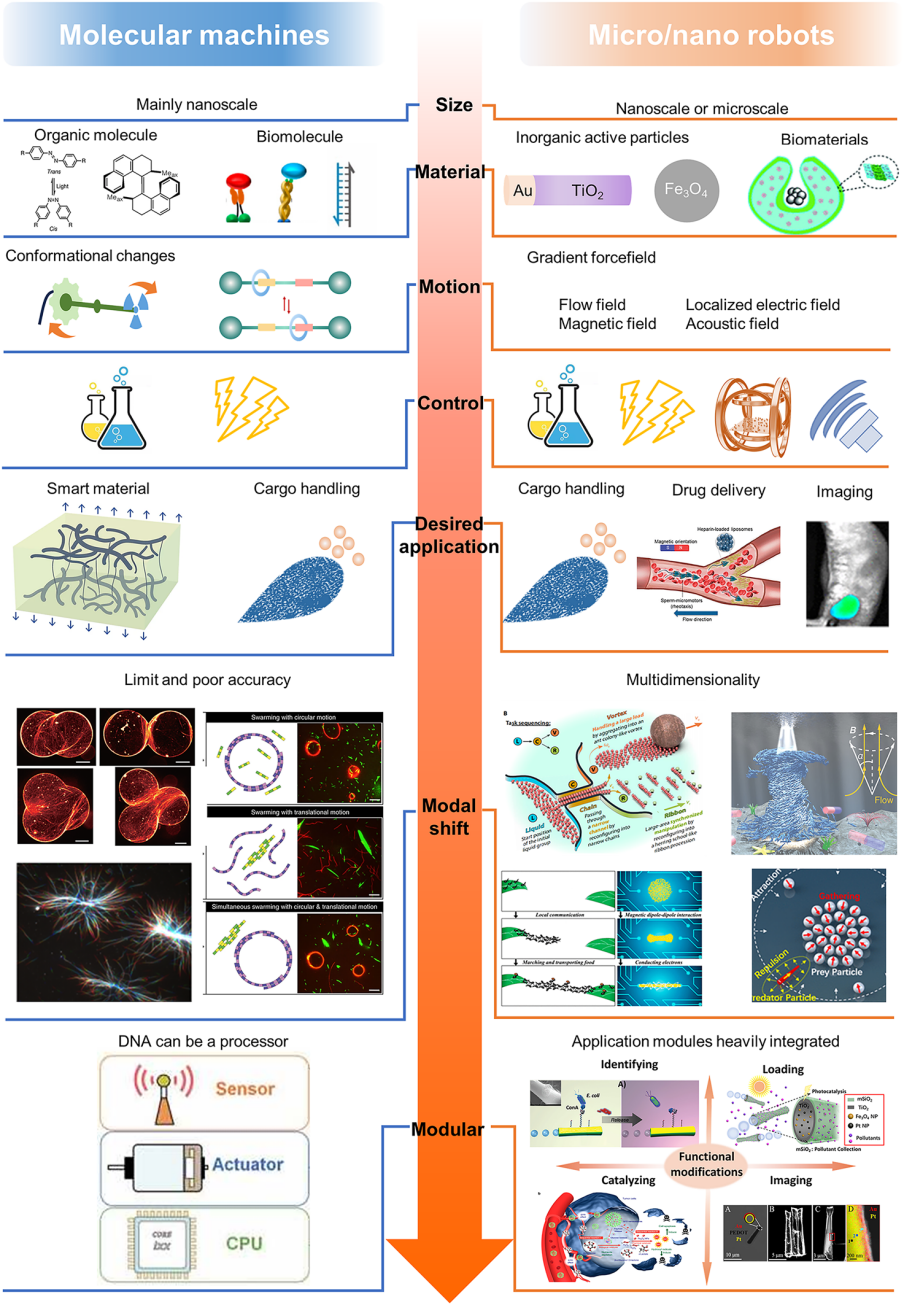
Molecular machines vs. micro/nano robots. In terms of size, one is mainly at the nanoscale, the other includes the micrometer and nanoscale. In terms of materials, one is mainly based on organic small molecules and biomolecules, the other is based on active particles, with biomaterials thrown in. In terms of mode of motion, one through conformational changes and the other through gradient force fields. In terms of control, one is dominated by chemistry and light, the other also includes magnetic fields and ultrasound. In terms of desired applications, one is used for building smart materials and micromanipulation, the other for micromanipulation, drug delivery and imaging. In terms of mode switching, molecular machines are currently more limited and less precise, compared to micro/nano robots that exhibit multi-dimensional and multi-layered structures in 2D, 3D and 4D. In terms of modules, molecular machines include receptors, controllers and actuators by virtue of the programmability of DNA. In contrast, micro/nano robots have integrated a large number of application modules, including recognition, loading, catalysis and imaging. Reprinted permission from Refs. [204–209]. Copyright @2019, American Chemical Society; @2020, American Chemical Society; @2012, American Chemical Society; @2018, American Chemical Society; @2018, Royal Society of Chemistry

It is essential to elucidate the disparities in design principles and behavioral patterns between molecular machines and micro/nanorobots for a more effective transfer of experience. These distinctions are illustrated in Fig. 8. Molecular machines primarily operate on the molecular or supramolecular scale, whereas micro/nanorobots can be slightly larger, extending to the micron scale. The smaller size of molecular machines facilitates intricate tasks, while larger micro/nanorobots are easier to design, modify, and integrate into collective behavior. From a material perspective, small organic molecules and biological macromolecules currently dominate molecular machines; hence chemical and light control mechanisms prevail due to their ability to induce chemical reactions. Conversely, micro/nanorobots are mainly composed of inorganic active particles, although many biological materials have been gradually added in recent years. This enables them to incorporate programmable physical fields such as magnetic fields and ultrasonics during control processes for achieving flexible collective behavior. The difference in motion mode is one of the most important differences in design principles between these two entities. Traditional molecular machines generate motion through conformational or configurational changes, while most micro/nanorobots do not undergo deformation during movement due to rigid nature of their inorganic components as well as adherence to the "scallop theorem" which favors gradient local fields over reversible deformations. The above differences at the individual level lead to differences in collective behavior to some extent. At this stage, compared to the rich collective modes of multi-dimensional, reconfigurable, and integrable micro/nanorobots, molecular machines are limited and have very low precision. The emergence of this difference will have the impact of design principles, but it does not mean that it is impossible to break through. In addition, molecular machines also have their advantages, because DNA provides powerful computing power to be able to act as a controller. In the content below, some insights and reflections on experience transfer will be provided.

First, the incorporation of active particles is expected to improve the precise control of molecular machine motion. Although molecular machines are capable of generating effective motion, slow and poorly controllable motion has always been a problem. In terms of the mode of actuation, micro/nanorobots are clearly more versatile in terms of the form of actuation, being able to move not only through the creation of concentration gradients [210–212], localized electric fields [213, 214], and fluid flow [215, 216], but also through the direct application of forces by magnetic fields [217] and ultrasound [218, 219]. More importantly, micro/nanorobots show better orientation. For example, the directional charge gradient induced by the direction of light allows the TiO_2_ microspheres to move along a specified path [214]. The magnetic field controls the directional movement of the clusters in the pipeline to reach the targeting site [209]. The constituent elements of the molecular machine then dictate that these small organic molecules and macromolecules are less susceptible to generating sufficient directional migration of electrons as in semiconductor materials, and even more difficult to produce magnetism. Combining active particles with molecular machines in order to achieve better motion control is expected. Some of the work has incorporated this idea. Materials that mimic magnetotropic bacteria have been reported [220]. Researchers encapsulated magnetic cobalt–platinum nanoparticles inside MTs, allowing the MTs to be efficiently magnetically aligned while maintaining motility. Similar work is expected, but there are issues in combining inorganic materials with organic materials, including and not limited to the maintenance of size, biocompatibility, and molecular stability.

In addition, the modal shift of molecular machines under the control of Multiphysics fields is yet to be achieved, which again can be drawn from micro/nanorobots. In terms of low dimensions, molecular machines already have embryonic collective behavioral patterns. This is mainly concentrated in the DNA and MT system, where MTs cluster and change their environment to form different clustering patterns, as mentioned earlier. In more flexible patterning and higher dimensional collective behavior has not yet been reported. All of these, a great deal of work has been realized in micro and nano robotics. For example, magnetic particles have the flexibility to switch between any of four modes: liquid, chain, vortex, and ribbon [221]. Through a combination of light and magnetic fields, magnetic micro swarms are able to overcome gravity to form tornado-like patterns [205]. The realization of these collective behaviors requires that the system be able to generate sufficient degrees of freedom. The degrees of freedom provided by the precise pairing of DNA is a strategy to address this, and this is one way that molecular machines advantage over micro/nanorobots. However, it is a challenge to rationally design the structure of a molecular machine to be able to utilize this degree of freedom. In addition, an effective combination of multiple control fields can also provide sufficient degrees of freedom. This can be done by combining active particles with the molecular machine, allowing the molecular machine to respond to multiple physical fields. However, this also requires solving the problems faced by combining molecular machines with inorganic materials. There is also a strategy of not designing the response unit of a control field such as a magnetic field directly on the molecular machine, but rather as an intermediate medium to convert the signal of the magnetic field into a chemical or optical signal perturbation, which in turn enables the molecular machine to respond.

Integrating more functional components, molecular machines are able to have more applications. Many application modules of micro/nanorobots come to perform multiple functions such as capture, loading, catalysis and imaging. These modules, effectively borrowed or ported over, could be what turns molecular machines into machines that are truly capable of performing multiple tasks. In addition to cargo delivery and capture of environmental pollutants, scheduling and control of catalysts with the help of molecular machines contribute to better catalysis. For example, for photocatalysis, where light is less permeable in solvent systems, molecular machines can assist in arranging catalysts in peripheral areas to better utilize light. In addition, with the clustered scheduling of molecular machines, finer 3D or 4D printing can be developed through methods such as magnetically controlled nesting and light controlled nesting. Further, such controllable mini-machines can also help in areas such as wearable sensing and drug delivery. All of these areas of exploration have significant challenges to overcome.

## Conclusion

In 1959, Feynman raised the possibility of building miniature machines on the nanoscale, but given the limitations of synthetic technology and other factors, this prescient idea was not realized until one could manipulate molecules [222]. The kinematic behavior of molecular machines is effectively amplified by collective control to produce lifelike motion and associated functions. As described in this progress report, the two modes of collective behavior are amazingly displayed in different scales. These integrated systems have touched a large number of applications ranging from energy storage devices to the construction of soft robots, and from cargo handling to micro/nano-scale manipulation. And in untouched areas, molecular machines may also be able to play a role in catalytic control and 3D printing. For example, changing the location and form of catalyst-substrate contact through swarming behavior. In addition, taking advantage of the multimodal transformation aspect of the swarm is expected to construct patterning and enable micro/nano-scale printing techniques.

This vision is wonderful, but it has to be acknowledged that the application of cluster behavior in molecular machines is only within the last decade. The precision of control, the strength of materials, and the flexibility of modal transitions have not yet matured. One can clearly imagine combining other types of molecular actuators in collective motions, such as conformationally stretchable helical chains, recognizable DNA building blocks, or reciprocally variable isomeric species. Such ideas could integrate various types of modules (gears, switches, and motors) that operate on their own mechanisms but communicate with each other. However, these are almost rarely addressed by work. Micro/nanorobots, despite their manifold differences from molecular machines, will certainly have aspects to learn from in the control of collective behavior. These experiences will help molecular machines to achieve enough strength and control precision to further constitute valuable collaborative work platforms through integration and modularization. Specifically, some clear "taking home" strategies are yet to be achieved. For example, macromolecular proteins or supramolecules are combined with inorganic particles such as Fe_3_O_4_ to achieve more precise control. Even more, this will lead to new response properties, such as magnetic fields. By introducing the control dimension of the environment or intermediate medium, such as the design of a special grating, the magnetic or ultrasonic signal into a chemical or optical signal disturbance, more physical fields can be introduced to enrich the control dimension. These are feasible not only for the design of responsive materials but also for the design of swarms to achieve multi-dimensional collective behavior from 1 to 4D similar to that of micro/nanorobots. In particular, molecular machines are able to utilize the precise pairing of DNA to generate collective processors, which is not the case with micro/nano robots [223]. This property can be used to construct heterogeneous swarms that mimic the compartmentalization of cells or pipelining to achieve an efficient and organic whole over the swarms. In addition, at this stage, the study of the two collective of molecular machines and micro/nanorobots behaviors seems to be not very connected, because they are oriented to different scales. Thinking about it differently, this is one of the advantages of being able to integrate them, since it is possible to design at both scales. This would facilitate the construction of multi-tasking autonomous entities composed of molecular machines.

In summary, we are excited about collective behaviors, but we must also acknowledge that these efforts are extremely challenging. This review hopes to provide insights into the work in this field. It is believed that this interesting field will attract more and more attention. With the rapid advances in synthetic chemistry, nanotechnology, biomolecular engineering, and artificial intelligence, it is expected that the major challenges mentioned above will be alleviated over time.

## Abbreviations

1D: One-dimension
2D: Two-dimension
3D: Three-dimension
4D: Four-dimension
AG: Azami-Green
AR: Aspect ratio
azo-LCNs: Azobenzene-functionalized liquid crystal polymer networks
CA: Collagen-actin
d-DNA: Dissociation DNA
DMSO: Dimethyl sulfoxide
GFP: Green fluorescent protein
LC: Liquid crystal
LCN: Liquid crystal network
l-DNA: Linker DNA
LMWG: Low molecular weight gelator
MOF: Metal–organic framework
MTs: Microtubules
pDNA: Photoresponsive DNA
PEG: Polyethylene glycol
r-DNA: Receptor DNA
TP: Tau-derived peptide
TP-AG: Tau-derived peptide-Azami-Green
UV: Ultraviolet
VIS: Visible
